# An immuno-epidemiological model for Johne’s disease in cattle

**DOI:** 10.1186/s13567-015-0190-3

**Published:** 2015-06-19

**Authors:** Maia Martcheva, Suzanne Lenhart, Shigetoshi Eda, Don Klinkenberg, Eiichi Momotani, Judy Stabel

**Affiliations:** Department of Mathematics, University of Florida, 358 Little Hall, Gainesville, FL 32611 USA; Department of Mathematics, University of Tennessee, Knoxville, TN 37996 USA; Department of Forestry, Wildlife and Fisheries, University of Tennessee, Knoxville, TN 37996 USA; Department of Farm Animal Health, Faculty of Veterinary Medicine, Utrecht University, Utrecht, 3584CL The Netherlands; Department of Human-care, Tohto College of Health Sciences, Tokyo Medical and Dental University, Fukaya, Saitama 366-0052 Japan; National Animal Disease Center, USDA, Ames, IA 50010 USA; Centre for Infectious Disease Control, National Institute for Public Health and the Environment, Bilthoven, The Netherlands

## Abstract

To better understand the mechanisms involved in the dynamics of Johne’s disease in dairy cattle, this paper illustrates a novel way to link a within-host model for *Mycobacterium avium* ssp. *paratuberculosis* with an epidemiological model. The underlying variable in the within-host model is the time since infection. Two compartments, infected macrophages and T cells, of the within-host model feed into the epidemiological model through the direct transmission rate, disease-induced mortality rate, the vertical transmission rate, and the shedding of MAP into the environment. The epidemiological reproduction number depends on the within-host bacteria load in a complex way, exhibiting multiple peaks. A possible mechanism to account for the switch in shedding patterns of the bacteria in this disease is included in the within-host model, and its effect can be seen in the epidemiological reproduction model.

## Introduction

Johne’s disease (JD) in dairy cattle is a chronic infectious disease in the intestines caused by the bacillus, *Mycobacterium avium* ssp. *paratuberculosis* (MAP). MAP in a contaminated environment infects cattle through oral route. Contaminated colostrum and milk from infected cows are important sources of infection for calves. Actual infection occurs when MAP bacilli are phagocytosed by M-cells covering the dome of the Peyer’s patches [1] and transported to macrophages. In the early stages of the infection, some of the MAP will not be destroyed by macrophages and will grow in those cells until cellular immunity will be generated. To develop specific cellular immunity, macrophages differentiate into epithelioid cells and then intracellular growth of the MAP will be suppressed. Epithelioid cells form specific structures, granulomas, which act to restrict MAP growth inside and destroy them gradually. Some of the MAP in the granuloma will survive and enter a period of dormancy until reactivation. Reactivation of MAP will start slowly in the subclinical stage in which intermittent shedding of MAP will start. Granulomatous enteritis develops during the subclinical stage and accelerates in the clinical stage. Histological studies of infected areas reveal the presence of many infected macrophages, but very little extracellular bacteria have been observed [2].

This disease exhibits a variety of shedding patterns of MAP into feces, usually with a brief initial shedding period and then followed by a long latent period. In later stages, some infected cattle progress to weight loss, diarrhea, and reduced milk production. The mechanisms of the pathogen and the reaction of the immune system that cause this long latent period are not well understood. Also the connection of underlying immunology responses and the variable shedding patterns is difficult to explain. See the following papers in this issue related to these mechanisms and their relationship with data on shedding patterns, the growth of granulomas, and the length of the latent period [3,4].

To better understand the mechanisms of this disease and to later choose control strategies, we will illustrate through modeling how the immune system of infectious cows may have an effect at the epidemiological herd level. In particular, we are interested in understanding how the host immune responses influence the epidemiological reproduction number of JD in a farm or a geographical region. We will study the complex immuno-epidemiology of JD through explicitly linking of epidemiological processes to the immune system dynamics.

Linking models at the two scales, immunological and epidemiological, has been done recently [5,6]. Different approaches have been used for such models, and some work has used decoupling assumptions to deal with the two scales separately [7-9]. In this paper, we are following the nested approach, introduced by Gilchrist, Coombs and Sasaki [10,11], in which the within-host model is independent of the between-host model but feeds into the between-host model. Our immuno-epidemiological model consists of two components: a time-since-infection dependent immunological model (within-host) and an epidemiological model (between hosts) whose transmission rates and virulence depend on the within-host variables. We will illustrate the basic ideas of linking a within-host model with a between-host model. For this illustration, we use a relatively simple model for the epidemiological dynamics. The representation of epidemiological reproduction number shows the dependence on specific within-host populations. Since there may be a type of switch in the within-host system that accounts for the variability of the shedding patterns, we give a way to illustrate a possible switching mechanism and show its effect on the epidemiological reproduction number. We use the stimulation rate of the immune response from infected macrophages as this switch.

In the next section, we introduce our within-host model, and then we discuss its estimated parameter values and stability analysis. We include a mechanism to switch from low bacterial load to high bacterial load. The third section gives the between-host model linked to the within-host model. We show how the epidemiological reproduction number can be represented in terms of the equilibrium points of the within-host model.

## Materials and methods

### A within-host immunological model of JD

We give some brief background of immunology of MAP leading to the within-host model.

### Essential immunology of MAP

MAP enters the body of a cow from environmental sources, including fecal material, at birth or early in life through milk. After entry, MAP travels to the intestines of the affected animal and infects the macrophages located in the Peyer’s patches [1]. In the early stages of the disease the bacterium prevents the macrophages from destroying it and the infected macrophages are kept dormant. The infection persists in the affected animal in the subclinical stage. Histological studies of infected areas reveal the presence of many infected macrophages but the amount of free bacteria has not been clearly documented. It appears that bacteria, that are released when an infected macrophage is destroyed, are immediately engulfed by new macrophages. To account for this observation, we include in the model uninfected macrophages and infected macrophages but no free bacteria. Immune responses to MAP are reviewed more thoroughly in [2] in this special issue. Early in the infection, a cellular immune response is activated through T cells and other cells. The cellular immune response is accompanied by proinflammatory cytokines, such as interferon-*γ* (IFN-*γ*) [12]. The cellular immune response is effective in controlling the infection, so that during the subclinical stage the shedding is often minimal or intermittent [13-16]. In the later stages of infection the cellular immune response wanes and a humoral response is activated in the form of B-cells and antibodies [17]. This response appears to be less effective in the controlling the infection and often increased shedding is observed at this stage of the infection. For this reason we include in the model the cellular immune response only.

### The immunological model

Within-host mathematical models describe the dynamics of cells that participate in the infection process within a single individual. In our system of differential equations with *τ* as the underlying variable for time since infection, we denote by *M*_*u*_ the number of uninfected macrophages in the infected area, *M*_*i*_ the number of infected macrophages in the infected area, and *C* the number of T cells in the infected area. The rate of change of uninfected macrophages increases by the total recruitment rate *r* of new macrophages to the area. Furthermore, it decreases by the new infections and by the uninfected macrophages’ per capita death rate *d*_1_. The uninfected macrophages become infected by the bacteria that come out of infected macrophages that are bursting. We represent this as direct transmission from infected to uninfected macrophages. To account for possible saturation as the number of infected macrophages increases, we incorporate per capita infection rate that levels off with the increase in *M*_*i*_. The proposed form of new infections per unit of time is a generalization of the mass action law, typically assumed in within-host models [18].

1 $$ \left\{\begin{array}{l}\frac{d{M}_u}{d\tau }=r-\frac{\alpha {M}_u{M}_i}{1+c{M}_i}-{d}_1{M}_u,\hfill \\ {}\frac{d{M}_i}{d\tau }=\frac{\alpha {M}_u{M}_i}{1+c{M}_i}-\frac{pC{M}_i}{1+{\varepsilon}_1{M}_i+{\varepsilon}_3C}-\delta {M}_i,\hfill \\ {}\frac{dC}{d\tau }=\frac{k{M}_i}{1+{\varepsilon}_2{M}_i}-{d}_2C,\hfill \end{array}\right. $$

The rate of change of infected macrophages increases by the rate of newly infected macrophages and decreases through the killing rate of cellular immunity and the death rate of infected macrophages *δ*. In the *M*_*i*_ differential equation, this double-saturation form is based on the assumption that one killer T-cell can kill only finitely many infected macrophages, independently of how many are present [19]. In the special case when *ε*_1_=0 and *ε*_3_=0, the killing rate is a mass action law. The per capita killing rate of T-cells is saturating in infected macrophages. Saturating force of infection and killing rates have been previously used in modeling human tuberculosis [20]. The rate of change of *C* is increasing in saturating fashion with respect to infected macrophages and is decreasing at per capita death rate of T-cells *d*_2_. We assume that in the presence of infected macrophages the immune response will be automatically activated, no additional conditions on the parameters are necessary. The dependent variables and the parameters are listed in Table 1.

**Table 1:** **Parameters and dependent variables and their interpretation**

**Variable or parameter**	**Meaning**
*M*_*u*_ (*τ*)	Number of uninfected macrophages at time *τ*
*M*_*i*_ (*τ*)	Number of infected macrophages at time *τ*
*C* (*τ*)	Number of immune cells at time *τ*
*r*	Total recruitment rate for uninfected cells
*α*	Infection rate of uninfected macrophages by infected macrophages
*d*_1_	Natural death rate for uninfected macrophages
*δ*	Death rate of infected macrophages
*d*_2_	Clearance rate for immune cells
*k*	Stimulation rate of immune response from infected cells
*ε*_1_	Reciprocal of half saturation constant for killing of infected macrophages
*ε*_2_	Reciprocal of half saturation constant for immune response stimulation rate
*ε*_3_	Reciprocal of half saturation constant for killing of infected macrophages
*c*	Reciprocal of half saturation constant for macrophages infection rate
*p*	Killing rate of infected macrophages by immune response.

### Fitting the immune model to calf data and parameter estimation

Estimating parameters in within-host models can be achieved through fitting to time series data and estimates of lifespans. We use calf data and procedures reported in [21,22]. Neonatal Holstein dairy calves were obtained from status level 4 herds with no reportable incidence of JD in Minnesota at 1–2 days of age. Calves were housed in Biosafety Level-2 containment barns for the duration of the study and experimentally inoculated by feeding milk replacer containing 2.6 × 10^12^ live MAP obtained by scraping the ileal mucosa from a clinically infected cow as previously described in [21]. Calves were dosed on days 0, 7, and 14 of the study. All procedures performed on the calves were approved by the Institutional Animal Care and Use Committee (National Animal Disease Center (NADC), Ames, Iowa). The clinical isolate of MAP was obtained from the ileum of a clinical cow at necropsy that had shed high numbers of MAP in the feces.

Infection of calves was determined by measurement of shedding viable MAP in the feces and recovery of MAP from tissues as described in [21]. After 12 weeks of incubation at 39 °C, viable organisms were determined by counting the number of colonies on the agar slants. IFN-*γ* and interleukin-10 (IL-10) were measured in cell-free supernatants to assess immune response to infection. Briefly, periperhal blood mononuclear cells (PBMCs) were isolated from the buffy coat fractions of whole blood and cultured at 2.0 × 10^6^/mL in complete medium at 39 °C in 5% *CO*_2_ in a humidified atmosphere. In vitro treatments consisted of no stimulation (medium only), concanavalin A (10 μg/mL), and a whole-cell sonicate of MAP (10 μg/mL) [22].

Macrophages have a lifespan of several months. Wigginton and Kirschner [20] uses three months for lifespans of both uninfected and infected macrophages. We set *d*_1_=0.25 months^−1^ and *δ*=0.3 months^−1^. We take a longer lifespan of infected macrophages because MAP prevents the self-destruction of infected macrophages [23]. We also fix *M*_*u*_ (0)=5and *M*_*i*_ (0)=2 so that they are consistent with the available data. We fit the remaining parameters to data. The data we use are colony forming unit (CFU) data and IFN-γ data for three calves (denoted by calf 9, calf 10 and calf 11). We complete one fit of the parameters for each data set for an individual calf. Since the model (1) does not have CFU or IFN-γ, we fit *c*_1_*M*_*i*_ (*τ*) to the CFU data and *c*_2_*C* (*τ*) to the IFN-γ data, where *c*_1_ and *c*_2_ are constants to be determined by the fitting. To reduce the number of parameters to be fitted we set *ε*_3_=0 and *ε*_1_=*ε*_2_. The parameter values are given in Table 2 which were obtained by fitting with Mathematica. Since we were fitting more parameters than data points, proper identification of the parameters was not feasible and potentially more than one set of parameters may match the data with good fit. However, the parameters in Table 2 are consistent with the available data and similar between the calves. They identify sensible parameter ranges for simulations to illustrate the key points of linking with the epidemiological model.

**Table 2:** **Estimated parameter values**

**Parameter**	**Calf 9**	**Calf 10**	**Calf 11**	**Units**
*r*	114	600	1425	cells/month
*α*	0.47	0.4	0.6	Month^−1^
*d*_2_	0.5	0.4	0.3	month^−1^
*k*	8.0	3.0	13.8	(cells × month)^−1^
*p*	3.8	1.4	16.1	(cells × month)^−1^
*ε*_1_	0.105	0.111	0.65	cells^−1^
*ε*_2_	0.105	0.111	0.65	cells^−1^
*ε*_3_	0.0	0.0	0.0	cells^−1^
*c*	0.0001659	0.0001	0.0001	cells^−1^
*C* (0)	26.3	15.3	0.1	cells
*c*_1_	0.005	0.01	0.01	unitless
*c*_2_	0.048	0.057	0.05	unitless

### Multiple equilibria and significance in terms of shedding

**Figure 1:**
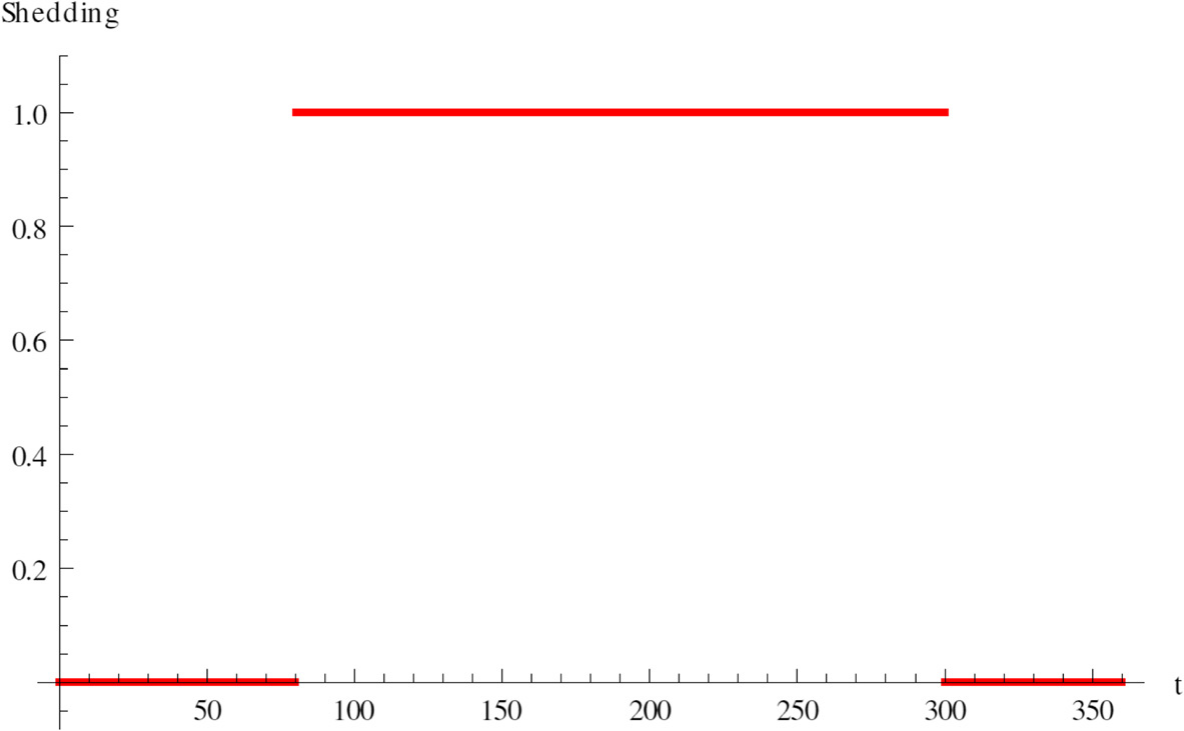
**Shedding pattern.** The x-axis shows time-post-infection in days. Non-shedding is plotted as zero, shedding as one.

One of the key features of JD in cattle is that animals, when infected, may switch between shedding and non-shedding, thus identified because shedding data are collected as a positive (shedding) or negative (non-shedding) status. As an illustration, the shedding pattern for one of the calves in our data-set is depicted in Figure 1. This figure shows that animals may spend a significant amount of time in each status, during which the shedding is relatively constant. Such a pattern is characterized by the presence of long, stable periods of indefinite lengths, and possibly multiple stable periods with switching between. Mathematically such dynamical behavior is captured by the presence of multiple steady states in the dynamical model, at least two of which should be locally stable (solutions that start close to them, converge to them). In this section we show that the immunological model (1) exhibits such multiple steady states.

To understand the presence and absence of steady states (equilibria) and how their stabilities change we need to define the immunological reproduction number

2 $$ {\mathrm{\Re}}_0=\frac{\alpha }{\delta}\cdot \frac{r}{d_1}. $$

The reproduction number gives the numbers of secondarily infected macrophages that one infected macrophage will produce in an entirely uninfected macrophage population during its lifespan as infected. Since JD is a chronic infection, we expect that for realistic parameter values ℜ_0_ > 1. Model (1) always has an infection-free equilibrium ${\mathrm{E}}_0=\left(\frac{r}{d_1},0,0\right)$, and if ℜ_0_ > 1, then there is at least one infected equilibrium *E*_*i*_=(*M*_u_^*^, *M*_i_^*^, ε_3_=0, C^*^). To find the equilibria we set the time derivatives in our system (1) equal to zero, resulting in the following equation in *M*_i_^*^:

$$ \frac{\alpha r}{\left({d}_1+\left({d}_1c+\alpha \right){M}_i^{\ast}\right)}=\frac{pk{M}_i^{\ast }}{\left({d}_2\left(1+{\varepsilon}_1{M}_i^{\ast}\right)\left(1+{\varepsilon}_2{M}_i^{\ast}\right)\right)}+\delta . $$

**Figure 2:**
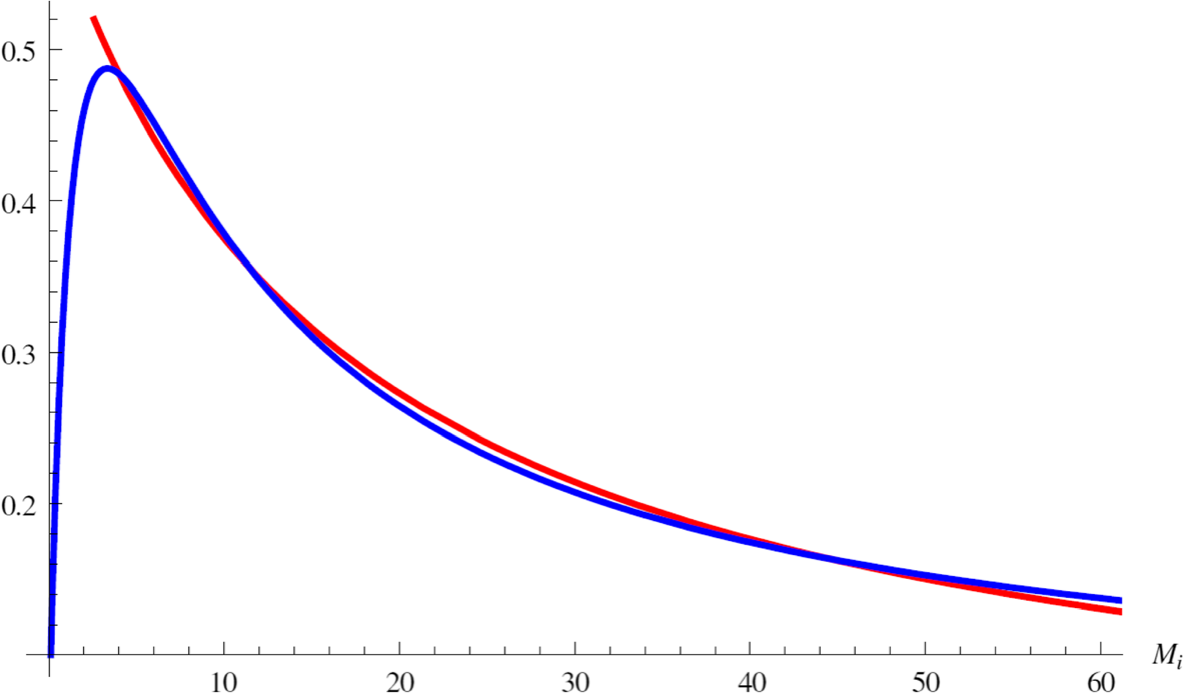
**Multiple equilibria.** Figure shows multiple intersections of the functions *f* (*x*) (in red) and *g* (*x*) (in blue). Each intersection gives one value of *M*_i_^*^ and therefore one equilibrium.

The solution *M*_i_^*^ of this equation gives us the infected equilibria. If *f* (*x*) denotes the left-hand side of this equation with *x* instead of *M*_i_^*^, and *g* (*x*) denotes the right-hand side, the solutions of the above equation are given by the intersections of the curves *y*=*f* (*x*) and *y*=*g* (*x*). It can be seen in Figure 2 that if ℜ_0_ > 1 for some parameter values, there could be three intersections when *M*_i_^*^ > 0. Each intersection gives one equilibrium value of *M*_i_^*^, and each equilibrium value of *M*_i_^*^ gives one equilibrium ${\mathrm{E}}_i=\left({M}_u^{*},{M}_i^{*},{C}^{*}\right)$, where

3 $$ {M}_u^{\ast }=\frac{r\left(1+c{M}_i^{\ast}\right)}{\left({d}_1+\left(\alpha +c{d}_1\right){M}_i^{\ast}\right)}\kern1em \mathrm{a}nd\kern1em {C}^{\ast }=\frac{k{M}_i^{\ast }}{d_2\left(1+{\varepsilon}_2{M}_i^{\ast}\right)} $$

**Figure 3:**
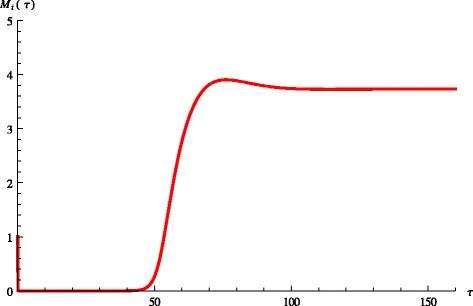
**Switch between equilibria.** Figure shows dynamic switch between the lower and upper equilibrium.

**Figure 4:**
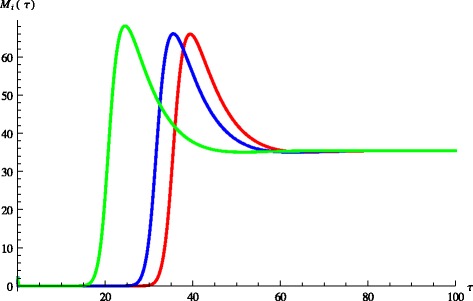
**Spontaneous switch between equilibria.** Figure shows dynamic spontaneous switch between the lower and upper equilibrium.

We label the equilibria in increasing value of *M*_i_^*^. It often can be mathematically shown that the lowest one is locally stable, the middle one is unstable and the upper one is also locally stable. In biological terms the presence of multiple stable equilibria allows animals to switch between low bacterial load and no shedding (below the detection limit) to high bacterial load and shedding and vice versa. If we assume that stressful events may temporarily affect the immune system negatively, we can model this temporary disturbance by modifying the constant parameter *k* (stimulation rate for immune response from infected cells) into a time dependent function:

4 $$ k\left(\tau \right)=\left\{\begin{array}{l}{k}_1\kern2em \tau \notin \left[{\tau}_1,{\tau}_2\right]\hfill \\ {}{k}_2\kern2em \tau \in \left[{\tau}_1,{\tau}_2\right]\hfill \end{array}\right. $$

where *k*_1_ > *k*_2_. This type of *k* (*τ*) may model for instance temporary reduction in the immune response strength due to stress. This minor disturbance may be very short-lived but may move the animal from a no-shedder to high-shedder, see Figure 3. Multiple equilibria do not occur for the values in Table 2 but for some modified values of the fitted parameters. However, in most cases for which *d*_1_=0.25 and *δ*=0.3, the lower equilibrium appears to be unstable while the upper one is stable. In this case the bacterial load and the shedding still exhibit a switch, however, the switch occurs spontaneously, without external disturbance. The length of the non-shedding phase depends on various factors, including the status of the immune system at infection. This scenario is illustrated in Figure 4.

### The immuno-epidemiological model of Johne’s disease

We imbed the immunological model (1) from the previous section into an epidemiological model of JD. The epidemiological model and the immunological model of JD are linked through the time-post-infection variable as well as the dependence of some epidemiological parameters on the within-host pathogen load.

### A brief description of JD epidemiology

JD is present in many countries with livestock industry and, in the United States, the causative agent of the disease, MAP, was found in about 70% of dairy farms [24,25]. After a long incubation time [26], dairy cattle infected with MAP start shedding the pathogen into feces, colostrum and milk [27-29]. MAP bacilli shed into feces can survive longer than a year in the environment [30] and the fecal-oral route was shown to be the major transmission pathway in dairy farms [31]. However, there is also evidence for transmission through contaminated milk, colostrum and placenta (vertical transmission) [29,31,32]. Young animals are more susceptible to MAP infection than adults [33], and therefore, good management practices to prevent MAP ingestion at young age (<1 year old) are suggested to be effective to control JD in dairy farms [34].

### The epidemiological component of the JD model

To account for differential susceptibility between calves and cows, we separate the the number of healthy calves *S*_*C*_ (*t*) from the number of healthy adults *S*_*A*_ (*t*), where *t* is the time (in months) since some initial point called time zero. Because the infectivity of infected individuals depends more on their shedding and pathogen load, rather than chronological age, we merge infected calves and infected adults in one class whose density with respect to time-post-infection is given by *i* (*τ*, *t*). The quantity *i* (*τ*, *t*) is a density since

$$ I(t)={\displaystyle {\int}_0^{\infty }}i\left(\tau, t\right)d\tau $$

is the number of all infected individuals at time *t*. The units of *i* (*τ*, *t*) are number of individuals per unit of time. The newly infected individuals move to the class *i* (0, *t*). The amount of bacteria in the environment is given by *B* (*t*). The model below has ordinary differential equations for *S*_*C*_, *S*_*A*_ and *B* and a first order partial differential equation for *i* (*τ*, *t*).

$$ \left\{\begin{array}{l}{S}_C^{\prime }(t)={b}_S\left(1-{\gamma}_C{f}_E(B)\right)+{\displaystyle {\int}_0^{\infty }}\left(1-q\left(\tau \right)\right){b}_I\left(\tau \right)i\left(\tau, t\right)d\tau \hfill \\ {}\kern8em -{\beta}_C{S}_C{\displaystyle {\int}_0^{\infty }}\kappa \left(\tau \right)i\left(\tau, t\right)d\tau -{\mu}_C{S}_C-a{S}_C\hfill \\ {}{S}_A^{\prime }(t)=a{S}_C-{\beta}_A{S}_A{\displaystyle {\int}_0^{\infty }}\kappa \left(\tau \right)i\left(\tau, t\right)d\tau -{\gamma}_A{S}_A{f}_E(B)-{\mu}_A{S}_A\hfill \\ {}\frac{\partial i}{\partial \tau }+\frac{\partial i}{\partial t}=-\nu \left(\tau \right)i-\mu i\hfill \\ {}i\left(0,t\right)={\displaystyle {\int}_0^{\infty }}q\left(\tau \right){b}_I\left(\tau \right)i\left(\tau, t\right)d\tau +{\beta}_C{S}_C{\displaystyle {\int}_0^{\infty }}\kappa \left(\tau \right)i\left(\tau, t\right)d\tau \hfill \\ {}\kern8em +{f}_E(B)\left({\gamma}_A{S}_A+{\gamma}_C{b}_S\right)+{\beta}_A{S}_A{\displaystyle {\int}_0^{\infty }}\kappa \left(\tau \right)i\left(\tau, t\right)d\tau \hfill \\ {}{B}^{\prime }={\displaystyle {\int}_0^{\infty }}\eta \left(\tau \right)i\left(\tau, t\right)d\tau - dB\hfill \\ {}\hfill \end{array}\right. $$

We assume that *b*_*S*_ gives the number of susceptible calves born per unit of time. A proportion *γ*_*C*_*f*_*E*_ (*B*) are infected soon after birth through contact with the infected environment. These move to the newly infected individuals *i* (0, *t*). Since newborn calves are separated after birth, we do not incorporate further environmental transmission of JD for calves. As a result of vertical transmission, from the number of calves born to infected individuals, a proportion 1–*q* (*τ*) are susceptible and remain in the class *S*_*C*_, while a proportion *q* (*τ*) are born infected and appear in the class *i* (0, *t*). The dependence of *q* on *τ* will be explained below. Susceptible calves can also become infected through direct contact with infected individuals (with coefficient *β*_*C*_) and also move to *i* (0, *t*). Susceptible calves progress to adulthood at rate *a*. Healthy adult cows can become infected through direct contact with infected individuals as well as through contact with the contaminated environment. In both cases the newly infected individuals move to *i* (0, *t*). We recall that calves are more susceptible to direct infection than adult cows. That fact implies that we need to have *β*_*C*_ > *β*_*A*_. Infected individuals shed bacteria into the environment at rate *η* (*τ*), and bacteria are cleared from the environment at a rate *d*. See Table 3 for the parameters and the dependent variables for the epidemiological model.

**Table 3:** **Parameters and dependent variables and their interpretation**

**Variable or parameter**	**Meaning**
*S*_*C*_ (*t*)	Number of healthy calves at time *t*
*S*_*A*_ (*t*)	Number of healthy adults at time *t*
*i* (*τ*, *t*)	Density of infected cattle with age-post-infection *τ* at time *t*
*B* (*t*)	Number of bacteria in the environment at time *t*
*b*_*S*_	Birth rate for susceptible cattle
*q* (*τ*)	Proportion of births of infected calves from infected cows
*β*_*C*_	Susceptibility of calves to infection due to milk
*μ*_*C*_	Natural death rate of healthy calves
*μ*_*A*_	Natural death rate of healthy adults
*μ*	Natural death rate of infected cattle
*a*	Rate of progression of calves to adulthood
*β*_*A*_	Susceptibility of adults to infection
*γ*_*C*_	Proportion of newborn calves getting infected from the environment
*γ*_*A*_	Rate of infection of healthy adults from the environment
*b*_*I*_ (*τ*)	Birth rate of infected adults *τ* units post infection
*κ* (*τ*)	Infectivity of infectious cattle *τ* units post infection

For the general function of environmental bacteria infectiousness *f*_*E*_ (*B*), we assume that 0 ≤ *f*_*E*_ (*B*) ≤ 1. We consider the following specific form of the function

5 $$ {f}_E(B)=\frac{1-{e}^{-\frac{B}{K_1}}}{1+{K}_2{e}^{-\frac{B}{K_1}}} $$

Where *K*_1_ and *K*_2_ are given constants. This function is chosen because it has a threshold effect: it is low for low values of *B* and at some threshold value of *B* increases fast to one.

### Linking the immunological and the epidemiological models

Understanding the impact of within-host pathogen dynamics on the spread of JD in a population of cattle requires proper linking of the epidemiological parameters to the within-host dynamic variables *M*_*i*_ (*τ*) and *C* (*τ*). The epidemiological parameters linked to the within-host parameters are the *τ*-dependent birth rate of infected cattle *b*_*I*_ (*τ*), the transmission rate of infected cows *κ* (*τ*), the disease-induced death rate *ν* (*τ*), the proportion of calves born infected $q\left(\tau \right)$ and the shedding into the environment *η* (*τ*). We assume that the cow’s birth rate decreases slightly with the progression of the disease as the weight loss and milk reduction lead to suboptimal conditions [35,36]. We model the effect of the disease-progression through the following relationship between birth rate and infected macrophage load:

$$ {b}_I\left(\tau \right)=\frac{b_S^{\prime }}{1+b{M}_i\left(\tau \right)} $$

where *b*^’^_*S*_ is the per capita birth rate of healthy cows and *b* has to be specified. The constant *b* controls the change in birth rate due to bacterial load and illness. Furthermore, the proportion of calves born infected also depends on the mother’s pathogen load and is represented by an increasing saturating function:

$$ q\left(\tau \right)=\frac{q{M}_i\left(\tau \right)}{Q+{M}_i\left(\tau \right)} $$

where *Q* is the half-saturation constant. The half-saturation constant controls how fast the curve reaches half-saturation level. Direct transmission rate *κ* (*τ*) is assumed to be small in JD. We surmise that it occurs when shedded feces become immediately infectious to susceptible calves or cows without having to stay in the environment and be subject to degradation. In this case the direct transmission rate is directly correlated with the CFU shed in the environment. Prior research suggests that in the case of HIV [37] the dependence of the transmission rate on the pathogen is saturating at high pathogen loads, and we use the simplest saturation function:

$$ \kappa \left(\tau \right)=\frac{M_i\left(\tau \right)}{\kappa_T+{M}_i\left(\tau \right)} $$

where *κ*_*T*_ is the half-saturation constant.

The disease-induced mortality is linked to the bacterial load as well as the immune response,

$$ \nu \left(\tau \right)=m{M}_i\left(\tau \right)+vC\left(\tau \right), $$

where *m* and *v* are scaling constants. The immune response leads to inflammation and thickening of the intestinal wall which causes diarrhea and malnutrition. Finally, the shedding into the environment *η* (*τ*) is given by the CFU and is taken to be proportional to the bacterial load:

$$ \eta \left(\tau \right)={c}_1{M}_i\left(\tau \right) $$

where *c*_1_ is estimated from fitting.

The linking above describes how the epidemiological parameters change with the within-host bacterial load. The resulting immuno-epidemiological model assumes that the within-host progression of the disease is averaged among all individuals and gives the population-level spread which results from the averaged immune dynamics.

### Reproduction number of the immuno-epidemiological model

Whether JD persists on epidemiological level depends on the epidemiological reproduction number R_0_ which gives the number of secondary infections one infected individual will produce in an entirely susceptible cattle population. If R_0_ > 1 then JD will persist in the population; if however, R_0_ < 1, JD may be eliminated from the population, even in the case when the infection persists in some individual infected animals.

To derive the epidemiological reproduction number, we first compute the disease-free equilibrium ${\mathrm{E}}_0=\left({S}_C^0,{S}_A^0,0,0\right)$ where

$$ {S}_C^0=\frac{b_s}{\mu_C+a}\kern2em {S}_A^0=\frac{a{b}_s}{\mu_A\left({\mu}_C+a\right)}. $$

The epidemiological reproduction number is then defined as:

6 $$ \begin{array}{l}{\mathrm{R}}_0=\left({\beta}_C{S}_C^0+{\beta}_A{S}_A^0\right){\displaystyle {\int}_0^{\infty }}\kappa \left(\tau \right)\pi \left(\tau \right)d\tau +\frac{\left({\gamma}_A{S}_A^0+{\gamma}_C{b}_S\right)f{\hbox{'}}_E(0)}{d}{\displaystyle {\int}_0^{\infty }}\eta \left(\tau \right)\pi \left(\tau \right)d\tau \hfill \\ {}\kern4em +{\displaystyle {\int}_0^{\infty }}q\left(\tau \right){b}_I\left(\tau \right)\pi \left(\tau \right)d\tau \hfill \end{array} $$

where

$$ \pi \left(\tau \right)={e}^{-{\displaystyle {\int}_0^{\tau }}\nu \left(\sigma \right)d\sigma }{e}^{-\mu \tau } $$

is the probability of survival in the infectious class. Each term of the reproduction number corresponds to a different mode of transmission of JD. The first two terms give the number of secondary infections of calves and adults who get infected through the direct transmission route. The third term gives the number of secondary infections, generated through environmental transmission. The integral in this term gives the amount of bacteria shedded by one infectious individual, 1/*d* gives the survival time of bacteria in the environment and *f*^′^_*E*_ (0) gives the rate of infection when the bacteria in the environment are rare. The last term gives the number of xsecondary infections generated through vertical transmission.

The epidemiological reproduction number depends on the immune system parameters through its dependence on *M*_*i*_ and *C*. It is instructive to see this dependence explicitly at least in the case when the immune system (1) is at equilibrium. In this case the solution of the system is given by ${\mathrm{E}}_i^{*}=\left({M}_u^{*},{M}_i^{*},{C}^{*}\right)$ where *M*_u_^*^ and C^*^ are given by (3). In this special case the epidemiological number becomes:

7 $$ \begin{array}{l}{\mathrm{R}}_0=\left[\left({\beta}_C{S}_C^0+{\beta}_A{S}_A^0\right)\frac{M_i^{*}}{\kappa_T+{M}_i^{*}}+\frac{\left({\gamma}_A{S}_A^0+{\gamma}_C{b}_S\right)f{\hbox{'}}_E(0)}{d}{c}_1{M}_i^{*}\right.\hfill \\ {}\kern4em +\left.\frac{q{b}_S^{\prime }{M}_i^{*}}{\left(1+b{M}_i^{*}\right)\left(Q+{M}_i^{*}\right)}\right]\frac{1}{m{M}_i^{*}+v{C}^{*}+\mu}\hfill \end{array} $$

### Estimation of epidemiological parameters

To illustrate the impact of within-host prevalence of MAP on the epidemiological reproduction number, we need estimates for some of the epidemiological parameters involved in the model. Reference [30] gives a detailed account of the survival properties of MAP in various environments. This allows us to estimate *d* which we take in the range 1/15–1/3 months^−1^. We also derive the shedding rate from the fits of the immune model to CFU data. The constant that links the MAP amount within a host *M*_*i*_ to the CFU is given by *c*_1_, and we set *c*_1_=0.01.

Reference [38] states that up to 25% of the calves may be infected in utero if the mother shows clinical signs. The removal rates *μ*_*C*_, *μ*_*A*_ and the age-progression rate *a* are well known for cattle [39]. The natural death rate *μ* is also known from [39]. The transmission parameters *β*_*C*_*, β*_*A*_, *γ*_*C*_, *γ*_*A*_ are assumed based on the expectation for an epidemiological reproduction number of about 2. The rate of infection of healthy adults from the environment can be obtained from [40]. The clearance rate of MAP *d* is taken from [30]. The epidemiological parameters are given in Table 4.

**Table 4:** **Parameters and dependent variables and their interpretation**

**Parameter**	**Meaning**	**Value**	**Range**
*b*_*S*_	Birth rate for susceptible cattle	100	0.1–1000
*b*_*S*_	Per capita birth rate for susceptible cattle	100	0.01–100
*b*	Constant in *b*_*I*_ (*τ*)	0.1	0.0001–1
*q*	Proportion of infected calves born to infected cows	0.25	0–0.25
*Q*	Half saturation constant	100	0–100000
*β*_*C*_	Susceptibility of calves to infection due to milk	0.0183333	0.001–1
*μ*_*C*_	Natural death rate of healthy calves	1/(10 × 12)	1/(25 × 12)–1/(5 × 12)
*μ*_*A*_	Natural death rate of healthy adults	1/(5 × 12)	1/(25 × 12)–1/(2 × 12)
*μ*	Natural death rate of infected cattle	1/(3 × 12)	1/(5 × 12)–1/(2 × 12)
*a*	Rate of progression of calves to adulthood	1/12	1/(2 × 12)–1/(0.5 × 12)
*β*_*A*_	Susceptibility of adults to infection	0.00733333	0.001–1
*γ*_*C*_	Proportion of newborns infected from environment	0.0001	0–1
*γ*_*A*_	Rate of infection of healthy adults from environment	0.0000183333	0.0–3
*κ*_*T*_	Half-saturation constant for *κ* (*τ*)	1000	10–10000
*m*	Proportionality constant for *ν* (*τ*)	0.3	0.0001–1
*v*	Proportionality constant for *ν* (*τ*)	0.01	0.0001–1
*η*_*E*_	Shedding rate of infected individuals	0.01	0.001–0.1
*d*	Clearance rate of MAP from the environment	1/5	1/15–1/3
*K*_1_	Constant in *f*_*E*_	1	1–10000
*K*_2_	Constant in *f*_*E*_	1	1–10000

## Results

### Implications of the within-host dynamics to the epidemiology of JD

**Figure 5:**
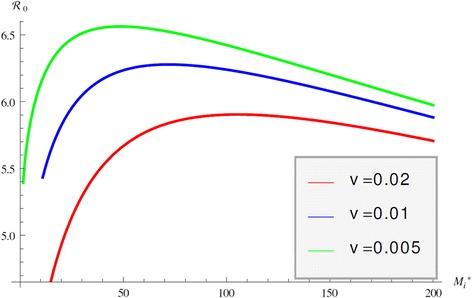
**R**_**0**_ **of direct transmission.** Figure shows the epidemiological reproduction number R_0_ as a function of the internal bacterial load *M*_i_^*^ when the only mechanism of transmission is direct transmission.

**Figure 6:**
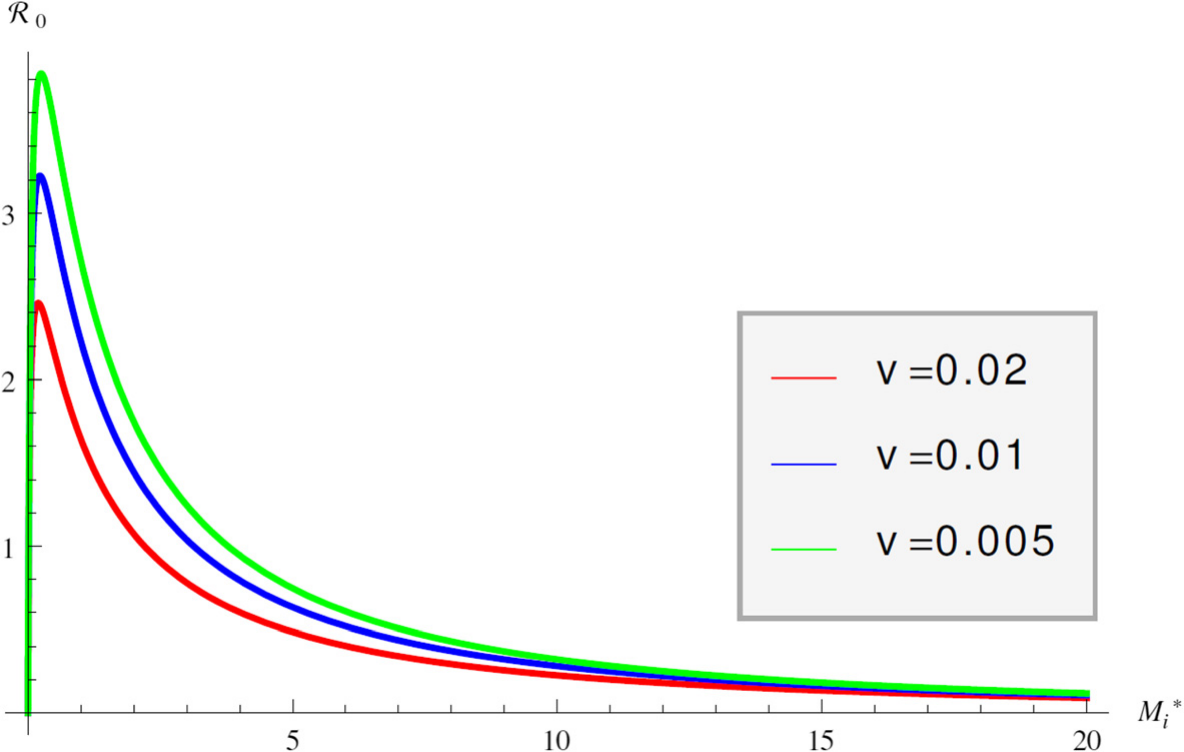
**R**_**0**_ **of vertical transmission.** Figure shows the epidemiological reproduction number R_0_ as a function of the internal bacterial load *M*_i_^*^ when the only mechanism of transmission is vertical transmission.

**Figure 7:**
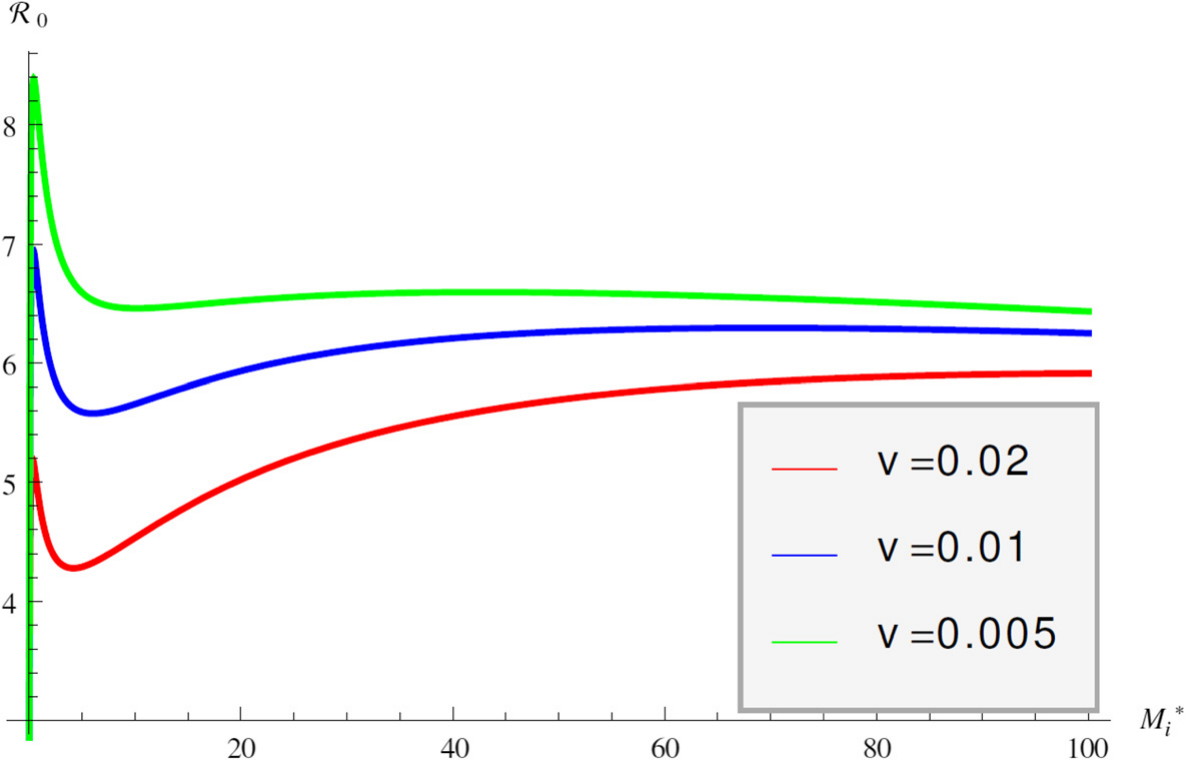
**R**_**0**_ **with all transmission modes.** Figure shows the epidemiological reproduction number R_0_ as a function of the internal bacterial load *M*_i_^*^ when all transmission modes are included.

Immuno-epidemiological models allow us to evaluate the impact of the within-host bacterial load on the epidemiological reproduction number. As we show, this dependence in the case of JD may be quite complicated. Intuitively, we may surmise that the epidemiological reproduction number is an increasing function of the bacterial load *M*_i_^*^. That is indeed the case when *M*_i_^*^ is small. When internal bacterial load is larger, then “trade off” effects come into play, and host mortality due to illness takes precedence. As a result the epidemiological reproduction number starts decreasing. This creates the typical “hump”-shaped form of the reproduction number as a function of the pathogen load [41]. We observe that shape in the epidemiological reproduction number of JD in the case when the transmission occurs only directly (*c*_1_=0, *q*=0) in Figure 5. Figure 5 also shows that the stronger the impact of the immune response on host mortality, that is the larger the *v*, the smaller the reproduction number. Furthermore, for larger *v* the peak in R_0_ is less pronounced and occurs for larger values of the bacterial load. This rather surprising effect that increasing *v* requires higher bacterial load for the maximum R_0_ to occur is produced by the saturation effect of the immune response in killing *M*_i_^*^ given by *ε*_2_. If *ε*_2_ is small the maximum occurs at smaller values for *M*_i_^*^ as *v* increases but as *ε*_2_ becomes larger, the immune response plays a smaller role. When transmission occurs only environmentally, that is *β*_*C*_*=β*_*A*_*=*0 and *q* = 0, the epidemiological reproduction number is an increasing function of *M*_i_^*^ and there is no maximum of R_0_ for intermediate values of *M*_i_^*^. We surmise that this is a result of the fact that shedding is directly proportional to the internal bacterial load and there is no saturation in the infection rate. When transmission occurs only vertically, then the epidemic reproduction number again rises for small internal bacterial load but reaches a peak quickly and then declines, see Figure 6. In this case the peak occurs for very small value of *M*_i_^*^. Furthermore, the weaker the impact of the immune response, the peak occurs for a larger value of *M*_i_^*^. When all three transmission mechanisms are combined, the reproduction number can experience several rises and declines, with several maximums for various values of the bacterial load *M*_i_^*^. For small values of *M*_i_^*^, vertical transmission dominates and leads to an early peak of R_0_ at small values of *M*_i_^*^. The survival trade-offs compensate, but as *M*_i_^*^ increases, the role of the vertical transmission declines and direct transmission takes on the dominant role, leading to a second peak in the reproduction number. Eventually the survival trade-off effect compensates for this increase also and R_0_ declines. The behavior of R_0_ with all three modes of transmission is given in Figure 7. This figure shows that as the effect of the immune system on the host *v* becomes smaller, the second peak becomes less pronounced and perhaps will disappear. To see the impact of the multiple within-host equilibria, we denote by *M*_*i*_^(1)^ the lower stable one and by *M*_*i*_^(3)^ the upper stable one. If the switch between the lower and the upper occurs at time *T*, we have

8 $$ {M}_i\left(\tau \right)=\left\{\begin{array}{ll}{M}_i^{(1)}\hfill & 0<\tau <T\hfill \\ {}{M}_i^{(3)}\hfill & \tau >T\hfill \end{array}\right. $$

This step-wise dynamics, approximates the actual dynamics which is continuous with a fast transition. We can define ${\mathrm{R}}_0^{(1)}$ and ${\mathrm{R}}_0^{(3)}$ to be given by (8) with *M*_*i*_ replaced by *M*_*i*_^(1)^ or *M*_*i*_^(3)^ respectively. Substituting (9) in the reproduction number (7), we obtain:

$$ {\mathrm{R}}_0={\mathrm{R}}_0^{(1)}\left(1-{e}^{-\left(m{M}_i^{(1)}+v{C}^{(1)}+\mu \right)T}\right)+{\mathrm{R}}_0^{(3)}{e}^{-\left(m{M}_i^{(1)}+v{C}^{(1)}+\mu \right)T}. $$

**Figure 8:**
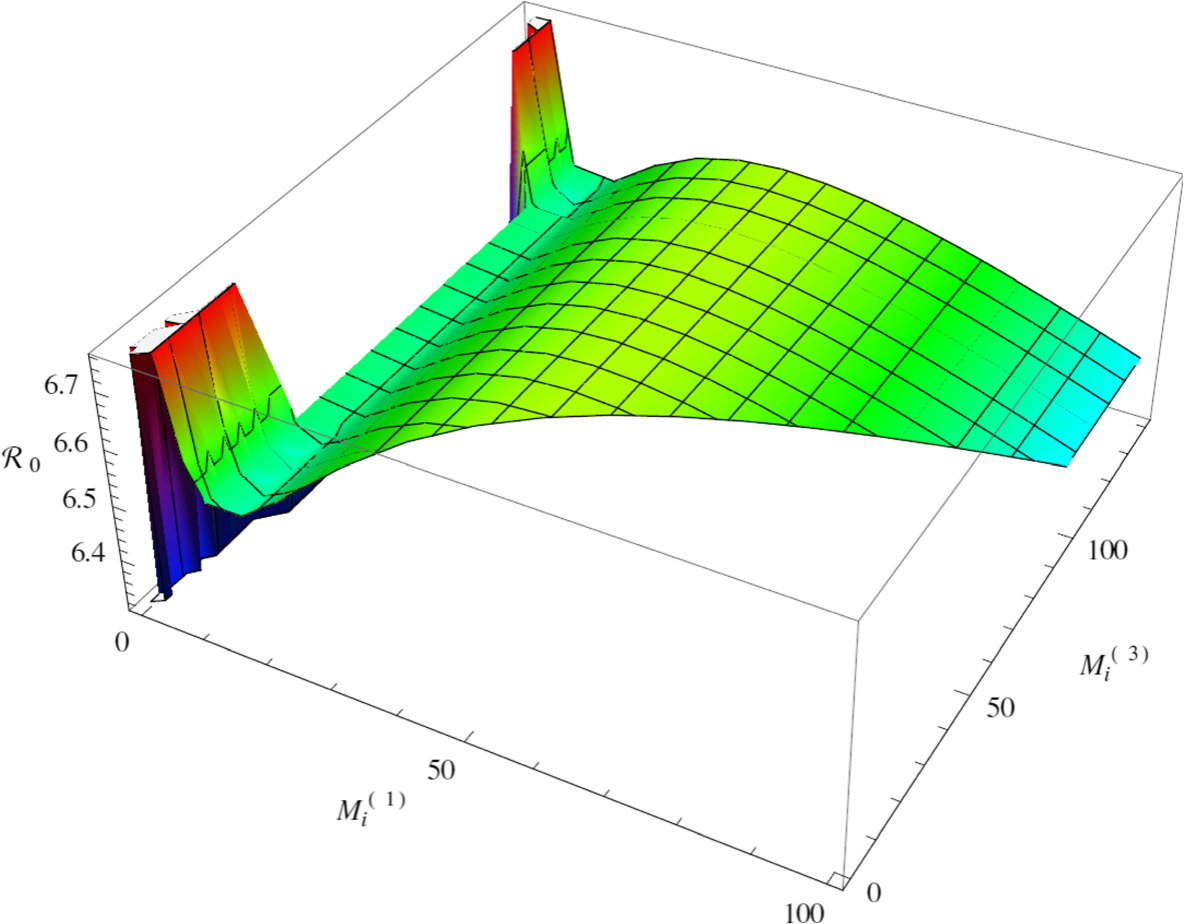
**R**_**0**_ **with switch in internal bacteria load.** Figure shows the epidemiological reproduction number R_0_ as a function of the internal bacterial load *M*_*i*_^(1)^ and *M*_*i*_^(3)^ when all transmission modes are included and switch time *T*=4.

From this expression, we can tell that if ${\mathrm{R}}_0^{(1)}$ < ${\mathrm{R}}_0^{(3)}$, then R_0_ is decreasing as *T* increases. However, this relationship between ${\mathrm{R}}_0^{(1)}$ and ${\mathrm{R}}_0^{(3)}$ is not guaranteed since the reproduction number could be large for small values of the within-host bacterial load and smaller for larger values of the bacterial load. We plot the reproduction number as a function of *M*_*i*_^(1)^ or *M*_*i*_^(3)^ with *M*_*i*_^(1)^ < *M*_*i*_^(3)^ and we see that it is changing dramatically (see Figure 8). Note that R_0_ is large for small *M*_*i*_^(1)^ but is very small for very large *M*_*i*_^(3)^. For slightly higher values of *M*_*i*_^(1)^ it sharply dips but then increases and subsequently decreases. Computing the prevalence in the case including all three modes of transmission is too complicated to be useful but typically prevalence increases with the increase of the reproductive number. That suggests that the prevalence may be high when most cattle appears nearly healthy and it may be decreasing when all infected cattle seems sick. This last fact is most likely due to increased culling in that case and reduced fertility.

## Discussion

Mathematical modeling is an important tool in learning about infectious diseases. We believe that linking immunological and epidemiological models will give further important contributions to understanding of multi-scale biological processes and to leading the way in disease control.

### Overview of insights of immunological model

The immunological model in the system describes the within-host dynamics of MAP. We fitted the model to CFU data of three calves. The fitting resulted in the estimation of a number of key quantities in the within-host dynamics of MAP. In particular we obtain values of the infection rate of uninfected macrophages, the stimulation rate of the immune response and others. These results are given in Table 2. The immunological model also reveals that saturating incidence in infected macrophages leads to multiple steady states of the bacterial load when ℜ_0_ > 1. In particular for some parameter regimes close to the estimated values, there are three nonzero equilibria, two of which are typically attracting. That allows for the MAP bacterial load to stabilize at two non-zero values, mimicking stable nonshedding and stable shedding. Switching between shedding and non-shedding can occur as a result of a short-term disturbance that a cow may experience. This suggests that prolonged no shedding, followed by prolonged shedding are two stable regimes where the switching occurs as a result of a stressor. Simulations show, however, that for some parameter regimes close to the estimated values, the lower equilibrium can be unstable and the switching between no shedding and shedding may occur spontaneously without any external disturbance. In this case the duration of the non phase depended on the initial infection and other factors.

### Overview of insights of immuno- epidemiological model

The epidemiological reproduction number R_0_, computed in terms of the within-host bacterial load, allows us to potentially infer when JD will persist or die out in a herd from the within-host bacterial load or shedding. At the very least, we can observe how the within-host bacterial load impacts the reproduction number, and hence, the prevalence of JD in a herd. JD is spread through three modes of transmission: environmental, vertical and direct. In general for directly transmitted diseases it is known that the epidemiological R_0_ exhibits a humped shaped dependence with respect to the pathogen load. Surprisingly, we find that the stronger the virulence caused by the immune response, the larger the pathogen load needed for the maximum to occur. Furthermore, we find that R_0_ also exhibits such a humped shape if the transmission is only vertical. In contrast with direct transmission, the peak in this case is more pronounced and occurs for lower pathogen load. The decline of R_0_ in both cases is attributed to the virulence. As JD is spread via all three modes of transmission, JD’s epidemiological R_0_ exhibits complex dependence on the within-host bacterial load with two peaks – the first one caused by vertical transmission and the second one caused by direct transmission. The non-monotone dependence of R_0_ on the within-host bacterial load does not allow us to infer in general that if the shedding of all cows in a herd is non-detectable, R_0_ and prevalence of JD will be low. In fact, no shedding (low pathogen load) in all cows may very well lead to high prevalence of JD in the herd. Because of that, control measures that reduce shedding or extend the time of the cows in a herd being non-shedders may not reduce the prevalence of JD.

### Significance of results

We introduce a new way to model long periods of shedding and no shedding as well as the switching between the two. This leads to understanding that these are due to stable stationary patterns of the within-host bacterial load. Furthermore, most likely the switching occurs due to temporary disturbance of the infected cow. Furthermore, the new model extends the modeling techniques to immuno-epidemiological models with multiple within-host equilibria and their impact on the epidemiology of disease. Finally, we uncover that in the case of multiple modes of transmission, as with JDs, the epidemiological reproduction number can depend on the pathogen load in a complex fashion, experiencing multiple peaks. Ultimately we conclude that no shedding (low pathogen load) does not necessarily imply low epidemiological reproduction number or low prevalence in the herd.

## References

[1] Momotani E, Whipple DL, Thiermann AB, Cheville NF (1988). Role of M cells and macrophages in the entrance of Mycobacterium paratuberculosis into domes of ileal Peyer’s patches in calves. Vet Pathol.

[2] Koets A, Eda S, Sreevatsan S (2015) The within host dynamics of Mycobacterium avium spp. paratuberculosis infection in cattle: where time and place matter. Vet Res (in press) 10.1186/s13567-015-0185-0 PMC4473847 26092382

[3] Klinkenberg D, Koets AP (2015) The long latent subclinical phase of Mycobacterium avium ssp. paratuberculosis infections explained without adaptive immunity. Vet Res (in press) 10.1186/s13567-015-0202-3 PMC4473850 26092036

[4] Magombedze G, Eda S, Bakker D, Koets AP (2015) Can immune response mechanisms explain the fecal shedding patterns of cattle infected with Mycobacterium avium subspecies paratuberculosis. Vet Res (in press) 10.1371/journal.pone.0146844 PMC4725749 26808389

[5] Martcheva M (2011). An immuno-epidemiological model of paratuberculosis. AIP Conference Proceedings.

[6] Numfor E, Bhattacharya S, Lenhart S, Martcheva M (2014). Optimal control applied in coupled within-host and between-host models. Math Models Nat Phenomena.

[7] Feng Z, Velasco-Hernandez J, Tapia-Santos B, Leite M (2012). A model for coupled within-host and between-host dynamics in an infectious disease. Nonlinear Dyn.

[8] Feng ZL, Velasco-Hernandez J, Tapia-Santos B (2013). A mathematical model for coupling within-host and between-host dynamics in an environmentally-driven infectious disease. Math Biosci.

[9] Ganusov VV, Bergstrom CT, Antia R (2002). Within-host population dynamics and the evolution of microparasites in a heterogeneous host population. Evolution.

[10] Gilchrist MA, Coombs D (2006). Evolution of virulence: Interdependence, constraints, and selection using nested models. Theor Popul Biol.

[11] Gilchrist MA, Sasaki A (2002). Modeling host-parasite coevolution: a nested approach based on mechanistic models. J Theor Biol.

[12] Begg DJ, de Silva K, Carter N, Plain KM, Purdie A, Whittington RJ (2011). Does a Th1 over Th2 dominancy really exist in the early stages of Mycobacterium avium subspecies paratuberculosis infections?. Immunobiology.

[13] Mitchell RM, Medley GF, Collins MT, Schukken YH (2012). A meta-analysis of the effect of dose and age at exposure on shedding of Mycobacterium avium subspecies paratuberculosis (MAP) in experimentally infected calves and cows. Epidemiol Infect.

[14] Wu CW, Livesey M, Schmoller SK, Manning EJ, Steinberg H, Davis WC, Hamilton MJ, Talaat AM (2007). Invasion and persistence of Mycobacterium avium subsp. paratuberculosis during early stages of Johne’s disease in calves. Infect Immun.

[15] de Silva K, Begg DJ, Plain KM, Purdie AC, Kawaji S, Dhand NK, Whittington RJ (2013). Can early host responses to mycobacterial infection predict eventual disease outcomes?. Prev Vet Med.

[16] Sommer S, Pudrith CB, Colvin CJ, Coussens PM (2009). Mycobacterium avium subspecies paratuberculosis suppresses expression of IL-12p40 and iNOS genes induced by signalling through CD40 in bovine monocyte-derived macrophages. Vet Immunol Immunopathol.

[17] Nielsen SS, Toft N, Okura H (2013). Dynamics of specific anti-Mycobacterium avium subsp. paratuberculosis antibody response through age. PLoS One.

[18] Nowak MA, May RM (2000). Virus dynamics: mathematical principles of immunology and virology.

[19] Gadhamsetty S, Maree AF, Beltman JB, de Boer RJ (2014). A general functional response of cytotoxic T lymphocyte-mediated killing of target cells. Biophys J.

[20] Wigginton JE, Kirschner D (2001). A model to predict cell-mediated immune regulatory mechanisms during human infection with Mycobacterium tuberculosis. J Immunol.

[21] Stabel JR, Palmer MV, Harris B, Plattner B, Hostetter J, Robbe-Austerman S (2009). Pathogenesis of Mycobacterium avium subsp. paratuberculosis in neonatal calves after oral or intraperitoneal experimental infection. Vet Microbiol.

[22] Stabel JR, Robbe-Austerman S (2011). Early immune markers associated with Mycobacterium avium subsp. paratuberculosis infection in a neonatal calf model. Clin Vaccine Immunol.

[23] Kabara E, Coussens PM (2012). Infection of primary bovine macrophages with Mycobacterium avium subspecies paratuberculosis suppresses host cell apoptosis. Front Microbiol.

[24] USDA/NAHMS report, Johne’s Disease on U.S. Dairies, 1991–2007. http://www.aphis.usda.gov/animal_health/nahms/dairy/downloads/dairy07/Dairy07_is_Johnes.pdf. Accessed 7 May 2015.

[25] Salem M, Heydel C, El-Sayed A, Ahmed SA, Zschock M, Baljer G (2013). Mycobacterium avium subspecies paratuberculosis: an insidious problem for the ruminant industry. Trop Anim Health Prod.

[26] Espejo LA, Godden S, Hartmann WL, Wells SJ (2012). Reduction in incidence of Johne’s disease associated with implementation of a disease control program in Minnesota demonstration herds. J Dairy Sci.

[27] Streeter RN, Hoffsis GF, Bech-Nielsen S, Shulaw WP, Rings DM (1995). Isolation of Mycobacterium paratuberculosis from colostrum and milk of subclinically infected cows. Am J Vet Res.

[28] Sweeney RW, Whitlock RH, Hamir AN, Rosenberger AE, Herr SA (1992). Isolation of Mycobacterium paratuberculosis after oral inoculation in uninfected cattle. Am J Vet Res.

[29] Sweeney RW (1996). Transmission of paratuberculosis. Vet Clin North Am Food Anim Pract.

[30] Elliott GN, Hough RL, Avery LM, Maltin CA, Campbell CD Environmental risk factors in the incidence of Johne’s disease. Crit Rev Microbiol (in press) 10.3109/1040841X.2013.867830 24670062

[31] Dore E, Pare J, Cote G, Buczinski S, Labrecque O, Roy JP, Fecteau G (2012). Risk factors associated with transmission of Mycobacterium avium subsp. paratuberculosis to calves within dairy herd: a systematic review. J Vet Intern Med.

[32] Whittington RJ, Windsor PA (2009). In utero infection of cattle with Mycobacterium avium subsp. paratuberculosis: a critical review and meta-analysis. Vet J.

[33] Windsor PA, Whittington RJ (2010). Evidence for age susceptibility of cattle to Johne’s disease. Vet J.

[34] Pillars RB, Grooms DL, Gardiner JC, Kaneene JB (2011). Association between risk-assessment scores and individual-cow Johne’s disease-test status over time on seven Michigan, USA dairy herds. Prev Vet Med.

[35] Smith RL, Strawderman RL, Schukken YH, Wells SJ, Pradhan AK, Espejo LA, Whitlock RH, Van Kessel JS, Smith JM, Wolfgang DR, Grohn YT (2010). Effect of Johne’s disease status on reproduction and culling in dairy cattle. J Dairy Sci.

[36] Smith RL, Grohn YT, Pradhan AK, Whitlock RH, Van Kessel JS, Smith JM, Wolfgang DR, Schukken YH (2009). A longitudinal study on the impact of Johne’s disease status on milk production in individual cows. J Dairy Sci.

[37] Lange A, Ferguson NM (2009). Antigenic diversity, transmission mechanisms, and the evolution of pathogens. PLoS Comput Biol.

[38] Seitz SE, Heider LE, Heuston WD, Bech-Nielsen S, Rings DM, Spangler L (1989). Bovine fetal infection with Mycobacterium paratuberculosis. J Am Vet Med Assoc.

[39] Lu Z, Schukken YH, Smith RL, Mitchell RM, Grohn YT (2013). Impact of imperfect Mycobacterium avium subsp. paratuberculosis vaccines in dairy herds: a mathematical modeling approach. Prev Vet Med.

[40] Magombedze C, Ngonghala CN, Lanzas C (2013). Evaluation of the iceberg phenomenon in Johne’s disease through mathematical modelling. PLoS One.

[41] Pugliese A (2011). The role of host population heterogeneity in the evolution of virulence. J Biol Dyn.

